# Effect of Perioperative Ketamine on Early Postpartum Depressive Symptoms Following Cesarean Section: A Meta-Analysis

**DOI:** 10.7759/cureus.107180

**Published:** 2026-04-16

**Authors:** Samantha Siu, Peter Hsin, Sarah Simon, Talia Lall, Anabel Lin, Da Young Lee, Rose Berkun, Patricia Junquera

**Affiliations:** 1 Department of Psychiatry and Behavioral Health, Florida International University, Herbert Wertheim College of Medicine, Miami, USA; 2 Department of Anesthesiology, Florida International University, Herbert Wertheim College of Medicine, Miami, USA; 3 Department of Anesthesiology, Jacobs School of Medicine, Buffalo, USA

**Keywords:** cesarean ​section, edinburg postnatal depressive score, esketamine, ketamine, meta-analysis, peri-operative medicine, postpartum depression

## Abstract

Postpartum depression (PPD) is a common condition associated with adverse maternal and infant outcomes. Higher rates have been observed following cesarean delivery, prompting interest in perioperative preventive strategies. Ketamine has demonstrated rapid antidepressant effects and may provide psychiatric benefits beyond analgesia. We conducted a systematic review and meta-analysis of randomized controlled trials evaluating perioperative ketamine or esketamine for early postpartum depressive symptom reduction in adults undergoing elective cesarean section. PubMed, ScienceDirect, and PsycINFO were searched for studies published between January 2015 and September 2025. Eligible studies reported Edinburgh Postnatal Depression Scale (EPDS) scores between postoperative days 3 and 14. A random-effects model was used to pool standardized mean differences (SMDs), with heterogeneity assessed using the I² statistic. Six randomized controlled trials met the inclusion criteria. Perioperative ketamine or esketamine was associated with a significant reduction in early postpartum depressive symptoms compared with control (SMD = −0.36; 95% CI −0.49 to −0.22). Moderate heterogeneity was observed (I² = 47.5%), with a consistent direction of effect favoring ketamine. These findings suggest that perioperative ketamine may reduce early postpartum depressive symptom burden, though further studies are needed to clarify optimal dosing, timing, and long-term clinical impact.

## Introduction and background

Postpartum depression (PPD) is one of the most common psychiatric complications of childbirth, affecting nearly 20% of new mothers worldwide [1]. Clinical features include depressed mood, anhedonia, irritability, guilt or worthlessness, and sleep disturbances, with diagnosis generally requiring symptom persistence for at least two weeks during pregnancy or within one year after delivery [2]. It is associated with substantial morbidity, including impaired maternal-infant bonding, delayed infant cognitive and emotional development, increased risk of maternal suicide, and long-term adverse effects on family functioning [2]. Despite its prevalence and clinical significance, preventive strategies for PPD remain limited, and current approaches largely rely on screening and treatment after symptom onset rather than prophylaxis.

Emerging evidence suggests that certain obstetric and perioperative factors may influence the risk of developing PPD. Cesarean section, in particular, has been associated with higher rates of postpartum depressive symptoms compared with vaginal delivery, potentially due to increased postoperative pain, delayed recovery, altered maternal expectations, and neurobiological stress responses [3,4]. These observations have prompted interest in perioperative interventions that may mitigate the risk of PPD, particularly among individuals undergoing elective cesarean delivery.

Racemic ketamine, a noncompetitive N-methyl-D-aspartate (NMDA) receptor antagonist, has traditionally been used intravenously for its anesthetic, analgesic, and opioid-sparing properties, whereas the S-enantiomer of ketamine, esketamine, has gained prominence as an intranasal treatment for treatment-resistant major depressive disorder and for reducing suicidal ideation [5]. These antidepressant effects are thought to involve modulation of brain signaling pathways that promote the formation of new neural connections and downstream neuroplastic changes [6].

Given these properties, perioperative ketamine administration presents a plausible strategy for reducing the risk of early postpartum depressive symptoms. Several randomized controlled trials (RCTs) and recent meta-analyses have examined perioperative ketamine or esketamine as a strategy to reduce postpartum depressive symptoms following cesarean section [7,8]. While many of these studies report statistically significant reductions in Edinburgh Postnatal Depression Scale (EPDS) scores during the early postpartum period, there remains uncertainty regarding the clinical interpretation of these findings [9]. Importantly, the EPDS used across these trials is a screening tool, not a diagnostic instrument for PPD. Thus, reductions in EPDS scores in the early postoperative period reflect changes in symptom burden rather than confirmed prevention of postpartum depression.

This study conducts a systematic review and meta-analysis of RCTs to evaluate the effect of perioperative ketamine administration on early postpartum depressive symptom severity, as measured by the EPDS, among adults undergoing elective cesarean section.

## Review

Methodology

Research Question

Does perioperative ketamine administration decrease the severity of postpartum depressive symptoms, as measured by the EPDS?

Inclusion Criteria

Population: Adults (≥18 years) undergoing elective cesarean section; Study design: RCTs; Intervention: Perioperative ketamine; Comparison: Control saline or standard anesthetic without ketamine; Outcome: Postpartum depressive symptoms between postoperative day (POD) 3 and POD14, measured using the EPDS.

Exclusion Criteria

Non-relevance: Manuscripts unrelated to perioperative ketamine administration in cesarean sections and PPD; Study design: Non-randomized studies including case reports, cross-sectional, prospective or retrospective cohort studies, non-randomized trials, qualitative studies, and systematic reviews; Language: Studies not available in English; Publication date: Studies published before 2015; Outcome reporting: Studies that did not report raw EPDS scores between POD3 and POD14; Additional exclusions: conference abstracts and trial registry entries due to insufficient outcome data for analysis.

Search Strategy

A systematic literature search, informed by PRISMA (Preferred Reporting Items for Systematic Reviews and Meta-Analyses) guidelines, was conducted across PubMed/MEDLINE, ScienceDirect, and APA PsycINFO to identify RCTs evaluating perioperative ketamine or esketamine for postpartum depressive symptoms. The search covered studies published between January 1, 2015, and September 24, 2025, using combinations of keywords including "ketamine", "esketamine", "postpartum depression", and "cesarean section". The search was limited to English-language human studies. In addition to database searches, reference lists of included articles were manually screened to identify any additional relevant studies.

Study Selection

**Figure 1:**
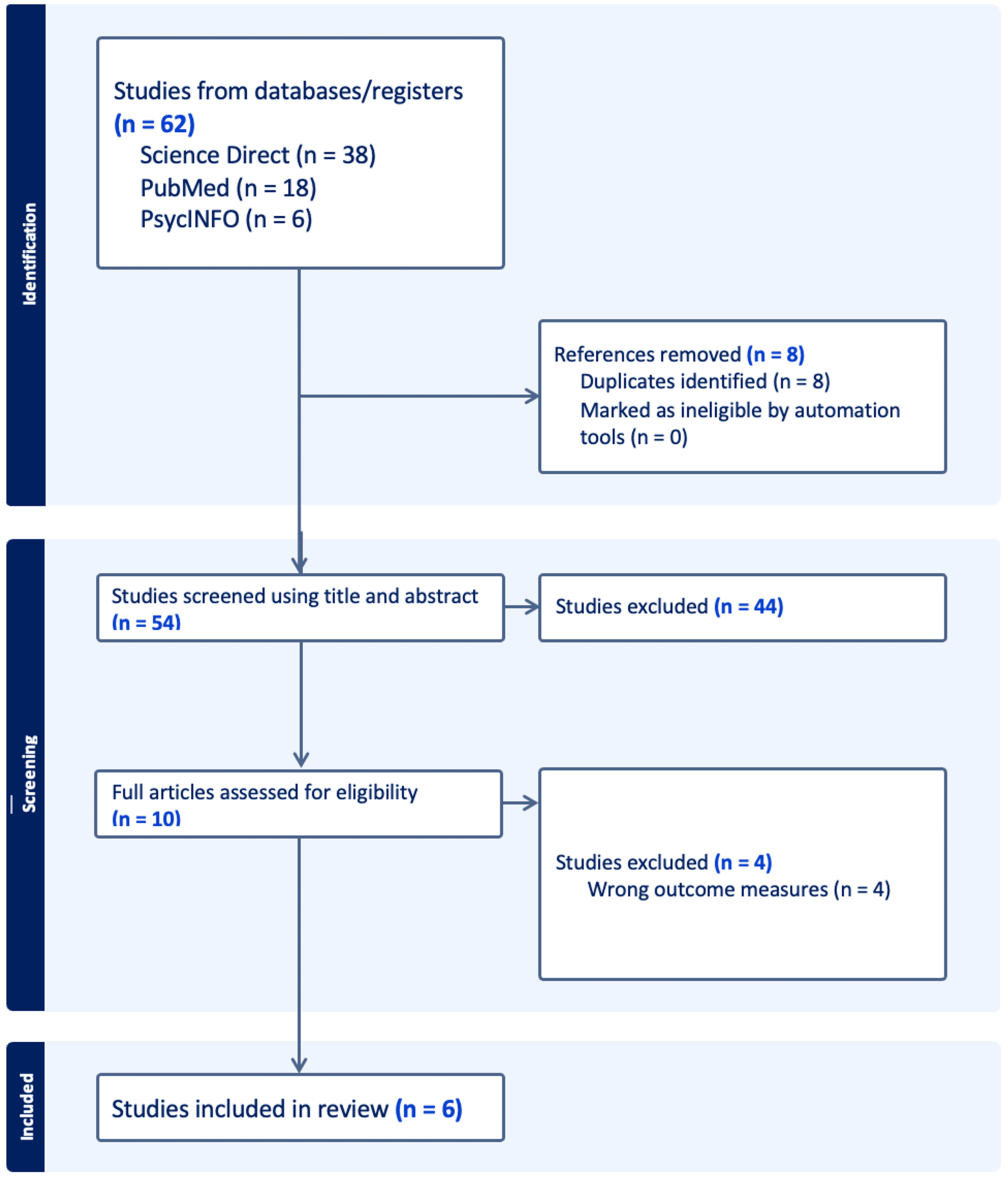
PRISMA Flow Diagram Depicting the Literature Search and Study Selection Process PRISMA: Preferred Reporting Items for Systematic Reviews and Meta-Analyses

Duplicate records were identified and removed using Covidence to ensure that each study was included only once in the analysis. All remaining records were screened independently by two reviewers in a two-stage process consisting of title/abstract screening followed by full-text review. At the full-text stage, eligibility was assessed independently by two reviewers, with disagreements resolved through discussion and consultation with a third reviewer. During the screening process, conference abstracts, study protocols, and clinical trial registry entries were also identified. However, these were excluded due to insufficient reporting of outcome data required for quantitative synthesis. The full selection process, including the number of records screened, assessed for eligibility, and included in the review, is summarized in the PRISMA flow diagram (Figure 1).

Study Characteristics

Six articles were included in the final sample for statistical analysis. Study characteristics were extracted from each study, including country, total sample size, ketamine dose, route of administration, and timing in relation to procedure, comparator, and outcome measurement. A summary of the study characteristics from the included trials can be found in Table 1.

**Table 1:** Study Characteristics of All Studies Included in Meta-Analysis EPDS: Edinburgh Postnatal Depression Scale; PCIA: Patient-Controlled Intravenous Analgesia Source: [10-15]

Study	Country	Total Sample Size (N)	Intervention (Dose, Route, Timing)	Comparator	Psychiatric Outcome Measure
Alipoor et al., 2021 [10]	Iran	134	Ketamine 0.5 mg/kg IV during induction	Thiopental (Nesdonal) induction	EPDS at 2 and 4 weeks post-op
Han et al., 2022 [11]	China	275	S-ketamine 0.5 mg/kg + sufentanil 2 µg/kg + tropisetron 10 mg as patient-controlled IV analgesia (PCIA) after delivery	Sufentanil 2 µg/kg + tropisetron 10 mg	EPDS at 3, 14, and 28 days post-op
Ma et al., 2019 [12]	China	654	Ketamine 0.5 mg/kg IV 10 min after delivery; PCIA (sufentanil 100 μg + ketamine 160 mg + palonosetron)	Standard postpartum care; PCIA (sufentanil 100 μg + palonosetron)	EPDS at 4–6 days and 6–8 weeks post-op
Wan et al., 2024 [13]	China	131	Esketamine 0.25 mg/kg IV single dose after umbilical cord clamping	Saline	EPDS at 3, 7, and 14 days post-op
Xu et al., 2017 [14]	China	330	Ketamine 0.25 mg/kg IV single dose within 5 minutes after cord clamping	Saline	EPDS at 3 days and 6 weeks post-op
Yao et al., 2020 [15]	China	308	Ketamine 0.25 mg/kg diluted to 5 mL with 0.9% saline IV single dose within 5 minutes after cord clamping	Saline	EPDS at 1 week, 2 weeks, and 1 month post-op

Quality/Risk of Bias Assessment

Only RCTs were included to minimize the risk of bias. Risk of bias for each study was assessed using the Cochrane Risk of Bias 2 (RoB 2) tool, evaluating bias arising from the randomization process, deviations from intended interventions, missing outcome data, outcome measurement, and selective reporting [16]. Two reviewers independently performed the risk of bias assessment, with a third reviewer available to resolve discrepancies. For deviations from intended interventions (Domain 2), the per-protocol effect was assessed, as most trials reported outcomes only for participants who completed the protocol.

Statistical Analysis

All statistical analyses and figure generation were performed using Microsoft Excel (Microsoft Corporation, Redmond, WA). Continuous outcomes were analyzed using the inverse-variance method. A random-effects model using the DerSimonian-Laird estimator was applied to account for between-study heterogeneity. Effect estimates were reported as standardized mean differences (SMDs) with 95% confidence intervals (CIs), generating an overall pooled SMD and corresponding p-value. PPD outcomes were assessed using EPDS scores. To standardize follow-up timing across studies, EPDS values were extracted at the timepoint closest to POD7, limited to POD3 through POD14. Statistical heterogeneity was evaluated using the I² statistic and τ². Given the limited number of included studies as well as variability in study design and reporting, no sensitivity analyses, subgroup analyses, or meta-regression were performed.

Publication Bias

Publication bias may be present, as only English-language studies reporting EPDS raw scores between POD3 and POD14 following elective cesarean sections with ketamine intervention were included. Given the small number of included studies (n=6), formal assessment of publication bias using funnel plot asymmetry was not performed, as such methods are unreliable with fewer than 10 studies.

Results

**Figure 2:**
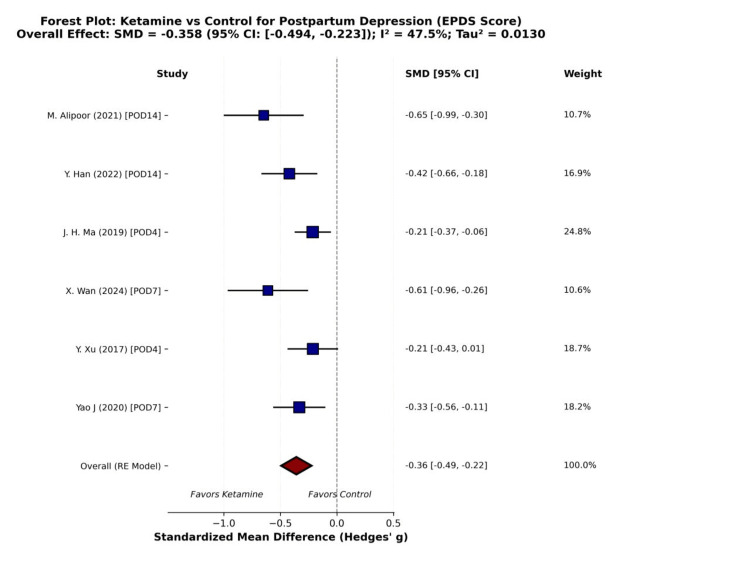
Effect of Ketamine vs. Control on EPDS Scores (POD 3–14) in Women Undergoing Elective Cesarean Delivery: Forest Plot POD: Postoperative Day; EPDS: Edinburgh Postnatal Depression Scale; SMD: Standardized Mean Difference; CI: Confidence Interval

Six RCTs met the inclusion criteria and were included in the quantitative meta-analysis. Using a random-effects model, perioperative ketamine or esketamine administration was associated with a statistically significant reduction in postpartum depressive symptoms, as measured by the EPDS, compared with control. The pooled effect size demonstrated a small-to-moderate benefit favoring ketamine (SMD = −0.36; 95% CI −0.49 to −0.22, p-value < 0.001). These results are illustrated in the forest plot (Figure 2).

Moderate heterogeneity was observed among the included studies (I² = 47.5%). The estimated between-study variance was low (τ² = 0.0130), indicating limited dispersion of true effect sizes across trials.

At the individual study level, most trials demonstrated an effect favoring ketamine. Alipoor et al. [10] reported the strongest reduction in depressive symptoms (SMD = −0.36; 95% CI −0.99 to −0.30), followed by Wan et al. [13] (SMD = −0.61; 95% CI −0.96 to −0.26), both indicating large effect sizes. Han et al. [11] demonstrated a moderate benefit (SMD = −0.42; 95% CI −0.66 to −0.18). Ma et al. [12] and Yao et al. [15] showed smaller but statistically significant reductions in EPDS scores (SMD = −0.21 and −0.33, respectively). Xu et al. [14] demonstrated a small effect size (SMD = −0.21), with a confidence interval crossing zero, indicating no statistically significant difference between ketamine and control in that study. A summary of the results is provided in Table 2.

**Table 2:** Summary of Meta-Analysis Results SMD: Standard Mean Difference; CI: Confidence Interval Source: [10-15]

Study	SMD	95% CI	Weight	Interpretation
Alipoor et al., 2021 [10]	-0.65	[-0.99, -0.30]	10.7%	Statistically significant medium effect favoring ketamine
Han et al., 2022 [11]	-0.42	[-0.66, -0.18]	16.9%	Statistically significant medium effect favoring ketamine
Ma et al., 2019 [12]	-0.21	[-0.37, -0.06]	24.8%	Statistically significant small effect favoring ketamine
Wan et al., 2024 [13]	-0.61	[-0.96, -0.26]	10.6%	Statistically significant medium effect favoring ketamine
Xu et al., 2017 [14]	-0.21	[-0.43, 0.01]	18.7%	Not statistically significant, small effect favoring ketamine
Yao et al., 2020 [15]	-0.33	[-0.56, -0.11]	18.2%	Statistically significant small effect favoring ketamine

**Figure 3:**
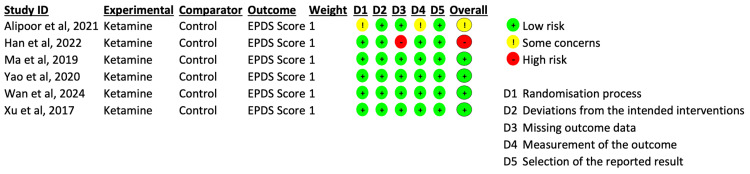
Risk of Bias Assessment Using the Cochrane Risk of Bias Tool (RoB 2) Risk of bias assessment across included studies [10-15] using the Cochrane Risk of Bias Tool (RoB 2) [16]. Domains assessed include bias arising from the randomization process, deviations from intended interventions, missing outcome data, measurement of the outcome, and selection of the reported result. Green indicates a low risk of bias; yellow indicates some concerns; and red indicates a high risk of bias.

Risk of bias was assessed using the Cochrane RoB 2 tool. The study by Alipoor et al. was rated as having some concerns due to incomplete reporting of methodological details [10]. The study by Han et al. was rated as high risk, as missing outcome data disproportionately affected participants in the ketamine group, potentially biasing the results [11]. All remaining studies were assessed as low risk using the RoB 2 tool, resulting in an overall low risk of bias across the included trials in this study. A summary of the risk of bias assessment is shown in Figure 3.

Discussion

In this meta-analysis of six RCTs, perioperative ketamine or esketamine exposure during cesarean section was associated with modestly significant lower EPDS scores compared to control between POD 3 and 14. The pooled SMD of -0.36 favored ketamine, indicating a small-to-moderate effect size.

From a clinical standpoint, an SMD of -0.36 suggests a modest reduction in depressive symptom burden rather than a dramatic preventive effect. While there is no universally established minimal clinically important difference for EPDS, prior studies have shown that a four-point absolute reduction is considered to be the EPDS’s Reliable Change Index (RCI), which is the difference between two scores required for a clinician to be 95% confident that there is a true change in the individual's mood [17]. Although this meta-analysis cannot definitively confirm that this threshold was reached across studies, even small reductions in EPDS scores may be meaningful. Subthreshold depressive symptoms and EPDS scores in the perinatal period are associated with an increased risk of subsequent PPD, so modest early improvements may still contribute to decreased emotional distress, improved maternal functioning, and reduced cumulative symptom burden over time [18].

Ketamine’s antidepressant effects are primarily attributed to NMDA receptor antagonism, which leads to enhanced glutamine signaling, increased brain-derived neurotrophic factor (BDNF) release, and downstream synaptogenesis in mood-related neural circuits [19]. These neuroplastic changes occur rapidly and may explain ketamine’s early antidepressant effects observed within days of administration. However, ketamine’s effects are not limited to glutamatergic signaling alone. Studies suggest that ketamine initially decreases GABA interneuron activity and NMDA signaling, but over time may normalize or even enhance GABA function, contributing to longer-term stabilization of neural networks and its antidepressant effects [20,21].

These mechanisms are particularly relevant to PPD, which is thought to involve dysregulation of inhibitory signaling after the abrupt withdrawal of reproductive hormones and neurosteroids, such as allopregnanolone, that normally enhance GABA-A receptor function [2,22]. Reduced GABAergic tone during the postpartum period has been implicated in mood instability and heightened stress sensitivity, which may contribute to postpartum depressive and anxiety symptoms [2]. Ketamine’s ability to influence GABA-glutamate interactions and restore network homeostasis can therefore be especially beneficial during this vulnerable period.

Current pharmacologic treatments for PPD further support the relevance of these neurobiologic pathways. Zuranolone, the first FDA-approved oral medication specifically for PPD, acts as a positive allosteric modulator of GABA-A receptors and is designed to restore inhibitory tone disrupted by postpartum neurosteroid withdrawal [23,24]. Although ketamine and zuranolone target different primary neurotransmitter systems (glutamate vs. GABA), both overlap in their abilities to restore stability in the neural circuits and inhibitory-excitatory balance. These findings support the hypothesis that modulation of network-level dysfunction, rather than individual neurotransmitter activity, may be central to effective treatment of PPD [23].

The included studies utilized intravenous formulations of ketamine, including both racemic ketamine and esketamine. Racemic ketamine contains both R- and S-enantiomers and has long been used in the United States as an intravenous anesthetic and analgesic in surgical and medical settings, whereas esketamine (S-ketamine) is most widely known as an intranasal, FDA-approved treatment for treatment-resistant depression [25]. Although these agents are often discussed interchangeably, distinctions between racemic ketamine and esketamine are clinically important due to differences in pharmacodynamics, routes of administration, regulatory statuses, and cost [25]. Additionally, intravenous racemic ketamine is also increasingly used off-label for depression, which has contributed to confusion regarding the differences between ketamine formulations [25]. With this context, the exclusive use of intravenous ketamine or esketamine in the studies included in this meta-analysis highlights the need for further research comparing various routes and formulations of ketamine in the psychiatric setting.

Limitations

Several limitations should be considered when interpreting these findings. Overall heterogeneity among included studies was moderate (I² = 47.5%), indicating that a substantial proportion of variability in effect sizes was attributable to between-study differences. Potential contributors include variation in ketamine dose, timing of administration, formulation, anesthesia protocols, baseline psychiatric risk, and timing of EPDS assessment.

Additionally, EPDS is a self-reported screening tool, and scores may fluctuate due to transient postpartum factors such as pain, sleep deprivation, and situational stress. Although EPDS demonstrates high sensitivity and specificity for major depression at scores ≥10, lower scores may still reflect clinically meaningful subthreshold symptom burden [26]. Furthermore, EPDS assessments were limited to postpartum days 3 through 14, capturing early depressive symptoms rather than formally diagnosed PPD. Given that PPD requires persistent symptoms for at least two weeks and may develop anytime within the first postpartum year, these findings cannot definitively establish the prevention of PPD, only an association with reduced early symptom severity [2].

Other limitations include the small number of included trials, which limited the ability to perform subgroup, sensitivity, and meta-regression analyses to further explore sources of heterogeneity. Notably, all included trials were conducted outside of the United States, limiting generalizability to U.S. peripartum populations and healthcare settings. While this review focused on randomized controlled evidence, it does not capture non-randomized studies, retrospective cohorts, or clinical practice experiences in which perioperative ketamine may have been administered and postpartum depressive symptoms assessed.

Methodological limitations related to the search strategy should also be considered. The literature search was restricted to English-language publications, which may introduce language bias and exclude relevant non-English studies. The use of a defined publication date range may have limited the inclusion of earlier studies, although this was intended to reflect contemporary perioperative and psychiatric practices. While multiple major databases were searched, it is still possible that relevant studies indexed elsewhere were not captured.

Future studies should prioritize standardized ketamine dosing protocols, longer follow-up periods extending beyond the early postpartum window, and clinically meaningful EPDS thresholds to better assess sustained antidepressant effects. Trials designed to distinguish preventive effects from short-term symptom reduction, as well as comparative studies between ketamine formulations and established PPD treatments, would further clarify ketamine’s role in peripartum mental health care.

## Conclusions

In this meta-analysis of RCTs, six studies examining the efficacy of perioperative ketamine administration during elective cesarean section in reducing postpartum depressive symptoms, as measured by EPDS scores between POD 3 and 14, were evaluated. Across these studies, perioperative ketamine was associated with a modest yet statistically significant reduction in EPDS scores. Moderate heterogeneity was observed among the included studies, warranting cautious interpretation of these findings, as heterogeneity may limit clinical applicability. These findings suggest that perioperative intravenous ketamine may represent a promising preventative strategy for PPD. Further well-designed randomized trials are needed to clarify its clinical utility, optimal dosing, and timing of administration.
